# Computer-assisted anteverting eccentric rotational acetabular osteotomy for recurrent posterior dislocation associated with acetabular retroversion: a case report

**DOI:** 10.1186/s13256-018-1949-8

**Published:** 2019-01-11

**Authors:** Hiroshi Imai, Jun Takeba, Akira Maruishi, Joji Miyawaki, Tomomi Kamada, Hiromasa Miura

**Affiliations:** Department of Bone and Joint Surgery, Ehime University Graduate School of Medicine, Shitsukawa, Toon, Ehime 791-0295 Japan

**Keywords:** Acetabular retroversion, Pincer-type femoroacetabular impingement, Posterior hip instability, Recurrent posterior dislocation of the hip, Eccentric rotational acetabular osteotomy, Preoperative three-dimensional surgical planning

## Abstract

**Background:**

Acetabular retroversion is a rotatory abnormality of the entire hemipelvis that includes anterior over-coverage and posterior deficiency of the acetabulum, and is associated with pincer-type femoroacetabular impingement and posterior hip instability. Acetabular retroversion is thought to cause posterior dislocation of the hip in athletes due to both the pincer-type femoroacetabular impingement and posterior hip instability.

**Case presentation:**

A 26-year-old Japanese man had acetabular retroversion that induced recurrent posterior dislocation of his hip due to excessive hip flexion while wakeboarding. We performed anteverting eccentric rotational acetabular osteotomy using preoperative three-dimensional planning and an intraoperative computerized navigation system. Our patient was able to return to sports activities 1 year postoperatively.

**Conclusions:**

Both preoperative three-dimensional surgical planning software and an intraoperative navigation system can provide a highly accurate map for this complicated surgery that simultaneously improves the pincer-type femoroacetabular impingement and posterior deficiency of the acetabulum.

## Introduction

Acetabular retroversion is a rotatory abnormality of the entire hemipelvis that includes anterior over-coverage and posterior deficiency of the acetabulum, and is associated with pincer-type femoroacetabular impingement (FAI) and posterior hip instability [1–4]. It is thought to cause posterior dislocation of the hip in athletes due to both the pincer-type FAI and posterior hip instability [3, 5–7]. Posterior dislocation of the hip is an orthopedic emergency because of the risk of avascular necrosis of the femoral head. Acetabular retroversion related to recurrent posterior dislocation of the hip is defined by a positive crossover sign, posterior wall sign, and ischial spine sign on an anteroposterior radiograph, and a hip with poor posterior acetabular support is defined by a posterior acetabular sector angle (PASA) of less than 90° on computed tomography (CT) [2, 3, 8, 9]. We experienced a case of acetabular retroversion that induced recurrent posterior dislocation of the hip due to excessive hip flexion while wakeboarding.

We performed anteverting eccentric rotational acetabular osteotomy (ERAO) for acetabular retroversion using preoperative three-dimensional surgical planning software and an intraoperative computerized navigation system. The patient was able to return to sports activities 1 year after the surgery. This report was approved by our institution’s scientific research board, and was conducted in accordance with the World Medical Association Declaration of Helsinki Standard of 1964, as revised in 1983 and 2000.

To the best of our knowledge, this is the first report to describe the treatment of recurrent posterior dislocation of the hip associated with acetabular retroversion using preoperative three-dimensional surgical planning software, an intraoperative computerized navigation system, and postoperative three-dimensional evaluation. And we believe in the importance of this operative planning and management in the treatment of recurrent posterior hip dislocation associated with acetabular retroversion.

## Case presentation

**Fig. 1:**
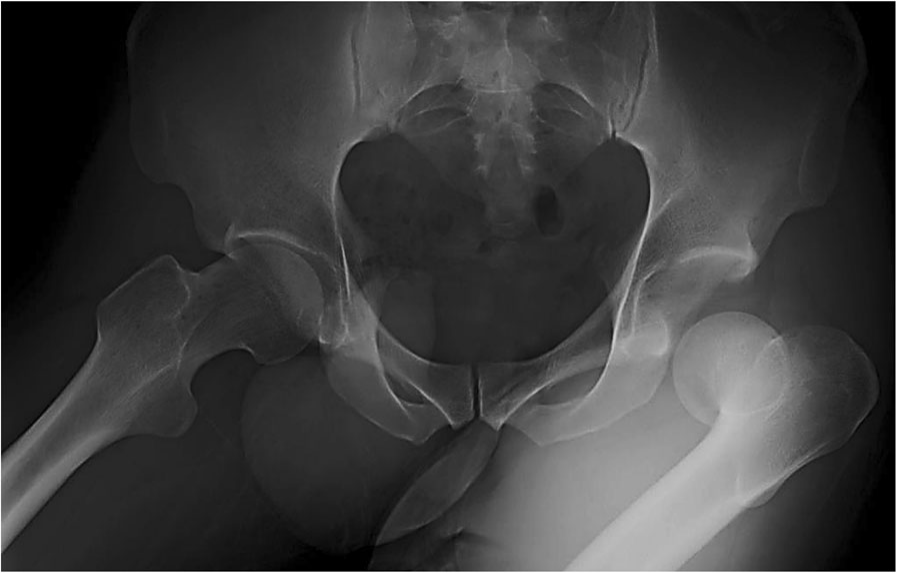
A plain radiograph shows an Epstein–Thompson type 1 posterior dislocation of the left hip with no fracture

A 26-year-old Japanese man was a previously healthy athlete with no prior hip joint problems. He was informed about the report in detail prior to providing written informed consent for enrollment, including consent for postoperative CT imaging. He fell off a wakeboard and impacted the water’s surface, causing excessive flexion of his bilateral hips and bilateral knees. He felt immediate and severe pain in his left hip, and was taken to a nearby hospital by ambulance. He had no signs of vascular injury or neurogenic deficits. A plain radiograph showed posterior dislocation (Epstein–Thompson type 1) of his left hip without fractures (Fig. 1) [10].

Closed reduction was performed under general anesthesia. A postoperative anteroposterior radiograph confirmed concentric reduction without joint space incongruity. A hip spica cast was used for 3 weeks to prevent hip dislocation recurrence. He was discharged from the hospital and returned to wakeboarding 3 months after the first dislocation. One year after the first injury, however, he sustained a second dislocation of his left hip, also while wakeboarding. He was taken to a hospital and a closed reduction was easily performed, but he subsequently experienced frequent posterior subluxation while wakeboarding or assuming a crouch position.

He was referred to our hospital for definitive treatment of recurrent dislocation of the hip. A physical examination showed that he was 174 cm tall and weighed 73.2 kg. Patrick’s test was negative and there was tenderness in Scarpa’s triangle. The anterior impingement sign was positive. The Japanese Orthopaedic Association (JOA) scores for evaluating hip joint function were 93 and 98 on the left and right, respectively. The JOA hip score is assessed using a 100-point scale that consists of the following subcategories: pain (0–40 points), ability to walk (0–20 points), range of motion (ROM; 0–20 points), and ability to complete daily living tasks (0–20 points). Higher scores indicate a better condition. Scores at the final follow-up were compared to the preoperative scores.

**Fig. 2:**
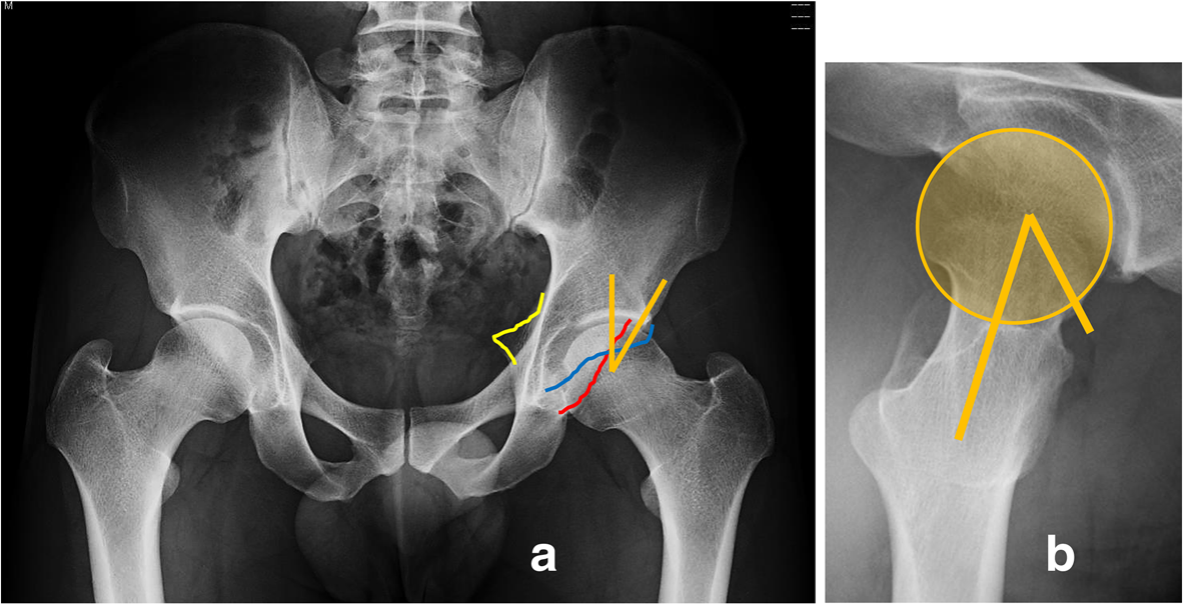
**a** Retroversion of the bilateral acetabulum is indicated by a positive crossover sign and a positive ischial spine sign. **b** The α angle on the left is 40°

Radiographic evaluation showed that the center-edge angle of both his left and right hips was 30° [11], and the α angles on the left and right were 43° and 40°, respectively [12]. There was retroversion of the bilateral acetabulum as indicated by a positive crossover sign [3] and a positive ischial spine sign (Fig. 2a, b) [2].

Three-dimensional CT scans were performed using a Philips Brilliance 64 scanner (Marconi Medical System, Best, The Netherlands). The scanning technique parameters were: 120 kV, 250 effective mAs, and a 0.5-second rotation time. Contiguous slices (2.0 mm) were obtained from the bilateral anterior superior iliac spines to the distal end of his femur, with our patient in a supine position, his hips extended, and thighs horizontal and parallel. All raw CT scan data were entered in a Digital Imaging and Communications in Medicine (DICOM) format into ZedHIP® planning software (LEXI Co., Tokyo, Japan) [13] and into a CT-based Hip Navigation System (Stryker Orthopaedics, Mahwah, NJ, USA).

CT scans revealed anterior over-coverage and posterior acetabular deficiency with an anterior acetabular sector angle (AASA) of 61° and a PASA of 85° [8, 9]. MRI images showed complete detachment of the capsulolabral complex from the posterior acetabulum.

**Fig. 3:**
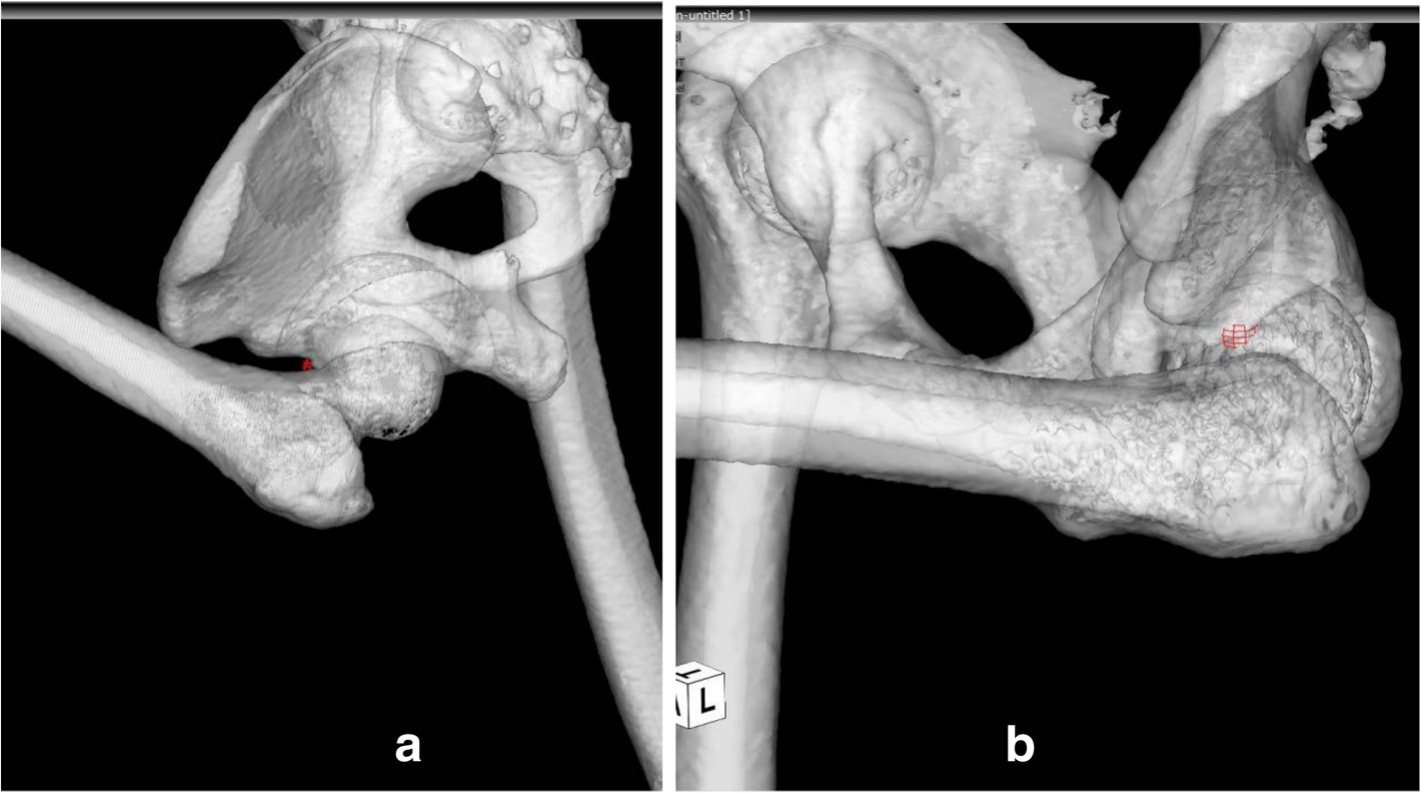
**a** The impingement angle between the anterior acetabular margin and the anterior portion of the femoral neck is 119° of flexion in the range of motion simulation. **b** The impingement angle between the anterior acetabular margin and the anterior portion of the femoral neck is 16° of internal rotation and 20° of adduction at 90° of flexion in the range of motion simulation

The angle at which impingement occurred in the ROM simulation was calculated by the ZedHIP® software based on CT images. The impingement angle between the anterior acetabular margin and the anterior portion of the femoral neck was 119° of flexion or 16° of internal rotation and 20° of adduction at 90° of flexion in the ROM simulation (Fig. 3a, b).

For the hip joint coordinate system [14], the plane that connects both anterior superior iliac spines and the pubic symphysis was defined as the anterior pelvic plane. The table-top plane was defined as the functional pelvic plane [15]. In addition, for the femoral coordinate system the reference plane for the femur was defined as follows: the plane that connects the most posterior points of the medial and lateral condyles and the most posterior point of the greater trochanter (in the table-top plane). The line connecting the piriform fossa of the femur and the center of the knee was defined as the femoral axis. The axis constructed by projecting the femoral axis to the femoral reference plane was defined as the *Z*-axis. The axis passing through the piriformis fossa perpendicular to the *Z*-axis and parallel to the formed plane was defined as the *X*-axis, and the cross-product of the *X*-axis and *Z*-axis was defined as the *Y*-axis.

Based on the clinical and radiographic examinations demonstrating acetabular retroversion accompanied by posterior wall deficiency, the causes of recurrent dislocation of our patient’s hip were considered to be anterior impingement between the anterior acetabular margin and the anterior portion of the femoral neck, as well as rupture of the posterior joint capsule.

**Fig. 4:**
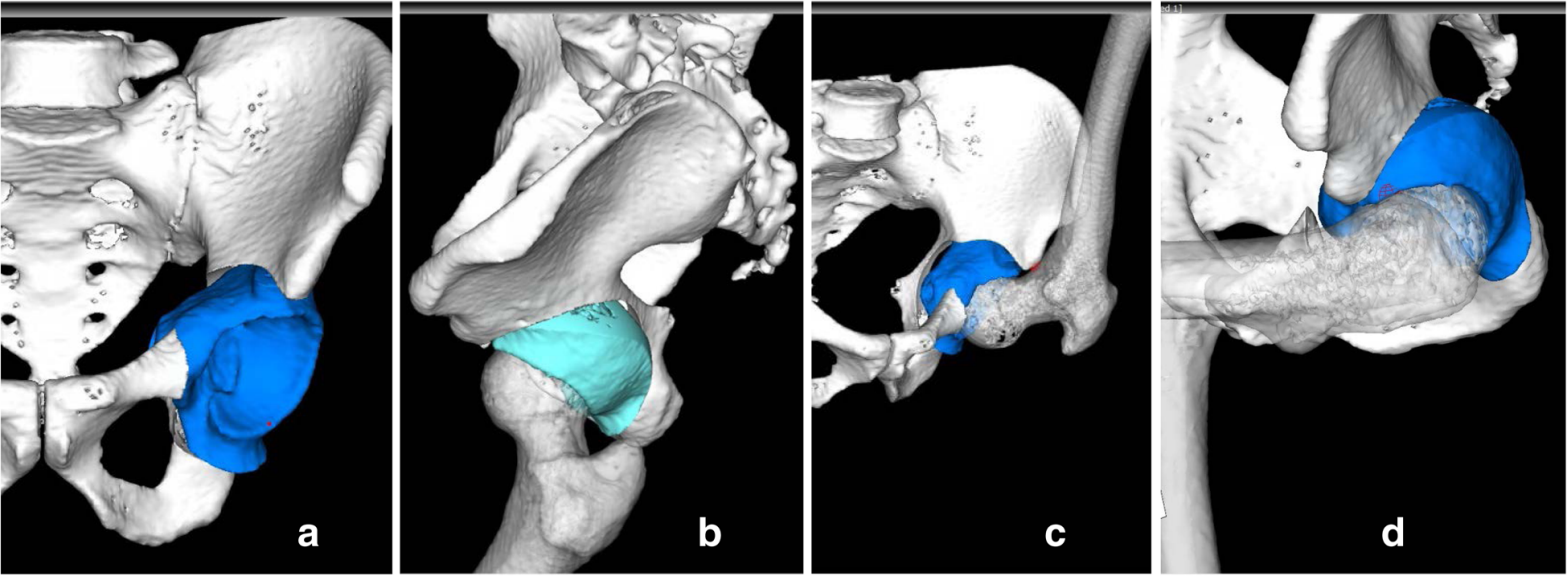
**a**, **b** We used the ZedHIP® software to plan moving the osteotomized fragment. **c**, **d** It could reach more than 125° of hip flexion or more than 20° of adduction and internal rotation at 90° of flexion

We performed conservative treatment for 3 months with a hip spica cast but an apprehension of a dislocation with excessive flexion or 30° of internal rotation at 90° of flexion of his left hip did not improve, so we scheduled operative treatment via anteverting ERAO. We used the ZedHIP® software to plan moving the osteotomized fragment so that it could reach more than 125° of hip flexion or more than 20° of adduction and internal rotation at 90° of flexion (Fig. 4a–d).

The anteverting ERAO was performed according to the technique described by Hasegawa *et al*. [16]. Our patient was positioned in the lateral position. A ball used by the infrared light navigation system was fixed with three pins onto the left iliac crest. The greater trochanter was detached with an oscillating saw and reflected proximally. The piriformis, obturator internus, and gemellus muscles were tagged and divided rather than torn. The posterior capsule was torn at the lower ischial femoral ligament.

**Fig. 5:**
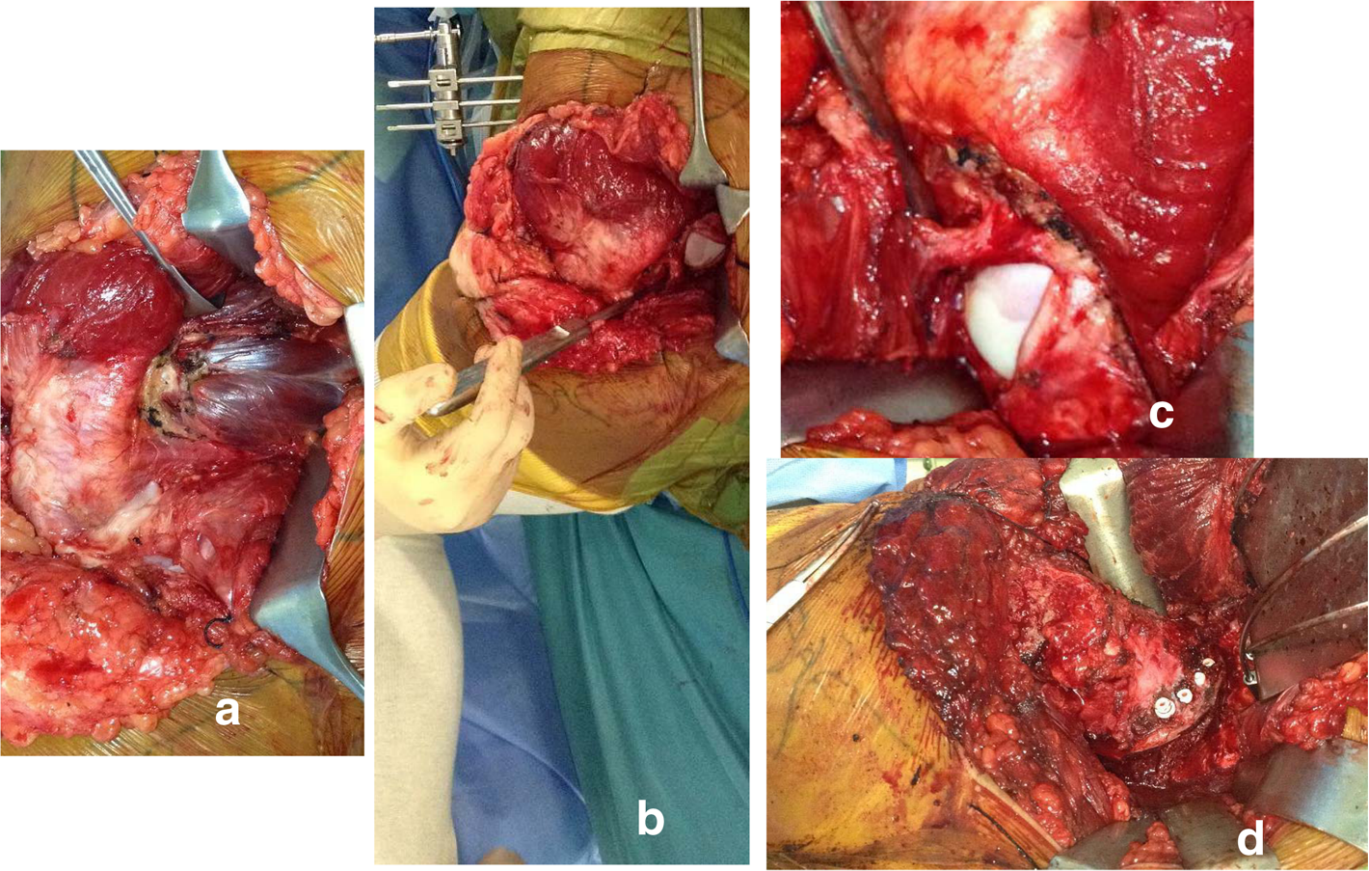
**a** The piriformis, obturator internus, and gemellus muscles were not torn. **b**, **c** The muscles were tagged and divided. The posterior capsule was torn at the lower ischial femoral ligament. **d** The planning angle and direction of the osteotomized fragment, both of which were calculated using the ZedHIP® software, were implemented intraoperatively using the CT-based Stryker Navigation System

**Fig. 6:**
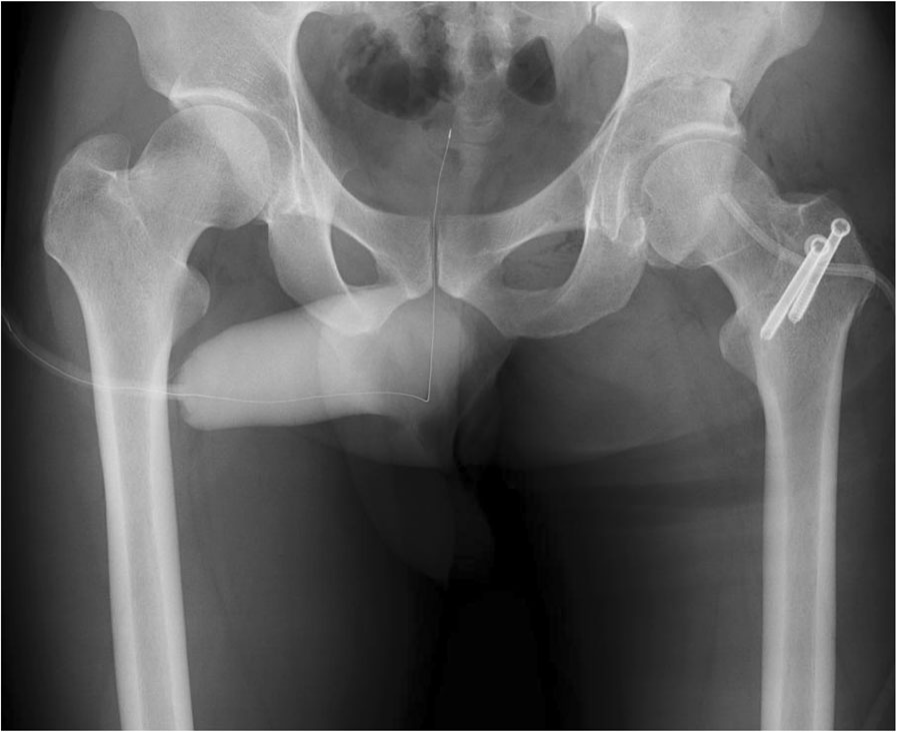
Intraoperative anteroposterior radiograph confirms the disappearance of the crossover sign and the presence of adequate posterior bone support

Posterior subluxation of the femoral head was defined when the hip joint reached more than 20° of adduction and internal rotation at 90° of flexion. A curved osteotomy chisel was introduced proximally 15 mm superior to the joint space, and anteverting ERAO was performed with a curved 45-mm-radius chisel. The planning angle and direction of the osteotomized fragment, both of which were calculated using the ZedHIP® software, were implemented using the CT-based navigation system during the operation (Fig. 5a–d). An intraoperative anteroposterior radiograph was also obtained, which confirmed the presence of adequate posterior bone support and the disappearance of the crossover sign (Fig. 6). Four hydroxyapatite screws were used to achieve fixation of the osteotomized fragment, and then the capsule and short rotator muscles were repaired. After fixation of the fragment, we verified that there was no tendency for the hip to sublux due to impingement by ensuring hip flexion of at least 120° or adduction and internal rotation of at least 20°, at 90° of flexion. When any subluxations due to impingement occurred, the osteotomy fragment was moved back into place. After verifying a lower rate of subluxation due to impingement, a drainage tube was placed and the wound was closed.

**Fig. 7:**
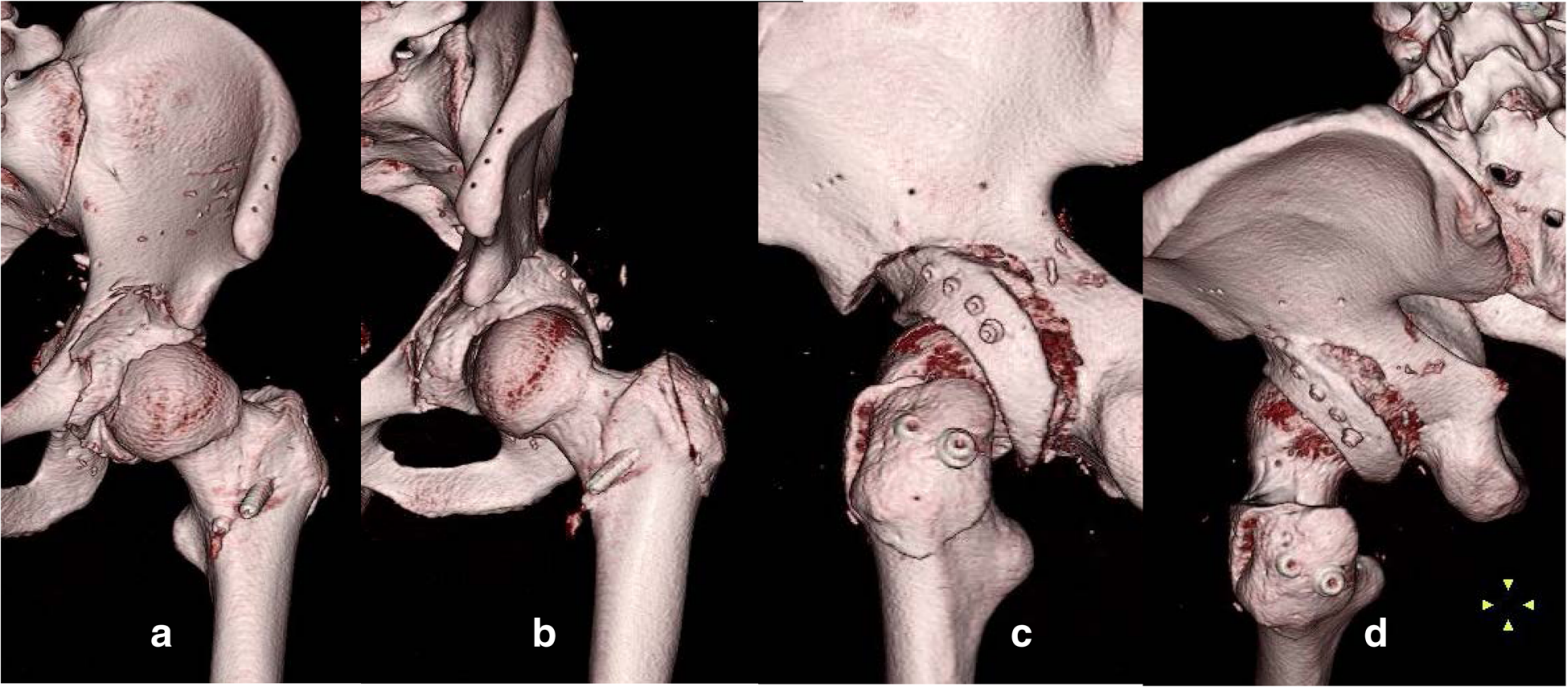
We performed three-dimensional computed tomography 2 weeks postoperatively. **a**–**d** Postoperative three-dimensional model of the pelvis

**Fig. 8:**
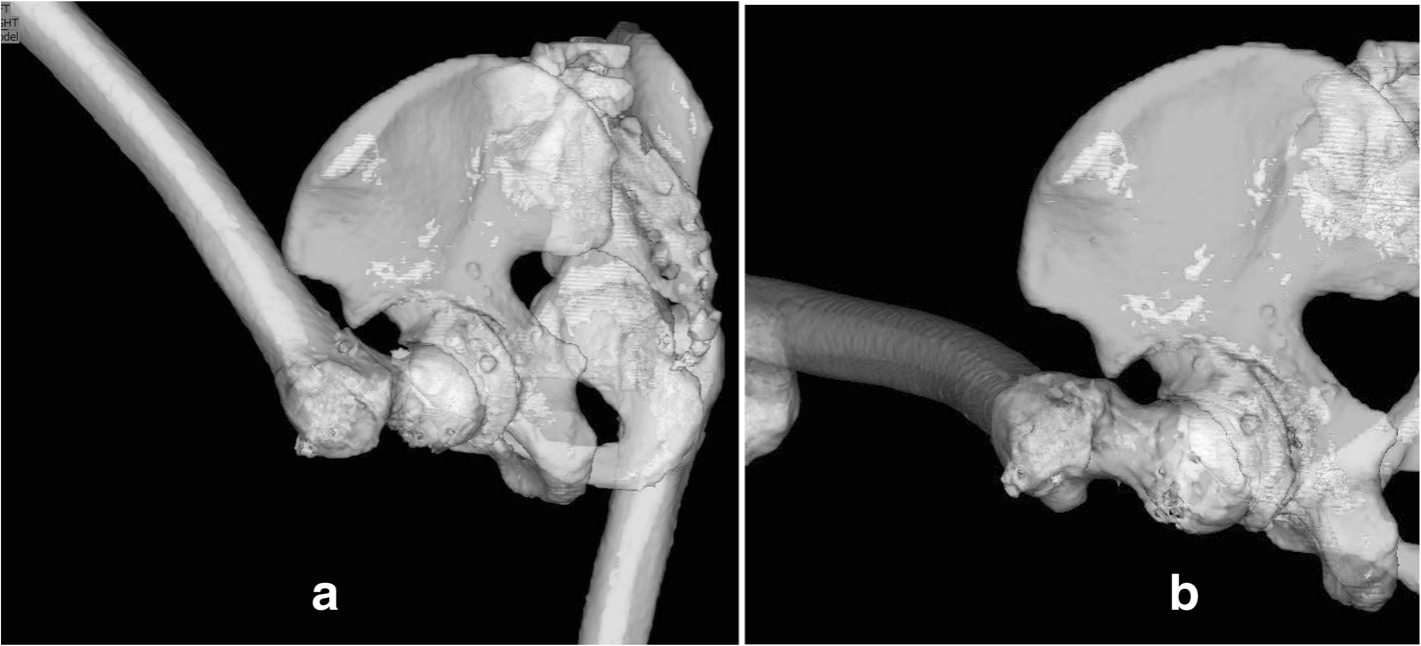
**a**, **b** Based on the postoperative CT images and using the ZedHIP® software, we assessed that this patient would be capable of at least 126° of hip flexion and at least 20° of adduction and 20° of internal rotation at 90° of flexion until impingement occurred between the anterior osteotomized fragment and the anterior portion of the femoral neck

On postoperative day 1, our patient was in good overall clinical condition. The drain was therefore removed and gait training with partial weight bearing was initiated. We performed a three-dimensional CT scan 2 weeks postoperatively (Fig. 7a–d). Based on the images obtained, the ZedHIP® software indicated that our patient was capable of at least 126° of hip flexion or at least 20° of adduction and at least 20° of internal rotation at 90° of flexion until impingement occurred between the anterior osteotomized fragment and the anterior portion of the femoral neck (Fig. 8a, b). The anterior impingement sign disappeared 3 months after surgery. Our patient gradually started to practice wakeboarding beginning 6 months after surgery, at which time the JOA hip score was 100 on the left side, and he returned to full wakeboarding activity at 1 year after surgery.

## Discussion

Acetabular retroversion is a rotatory abnormality of the entire hemipelvis. Anterior over-coverage and posterior deficiency of the acetabulum are present, and the condition is associated with pincer-type FAI and posterior hip instability. Posterior dislocation is not uncommon, and when it occurs it is an orthopedic emergency as it may cause avascular necrosis of the femoral head. Therefore recurrent posterior dislocation of the femoral head must be avoided if at all possible. Weber and Ganz discussed various treatments for recurrent dislocation of the hip associated with acetabular retroversion, including operative intervention to repair the hip joint capsule and posterior shelf osteoplasty [17]. Townsend *et al.* described the use of a posterior bone block to manage three patients with posttraumatic recurrent hip dislocations, two of whom had acetabular dysplasia [18]. Siebenrock *et al*. reported that anteverting periacetabular osteotomy for acetabular retroversion led to favorable long-term results with preservation of the native hip at a mean of 10 years, and significant improvements with regard to hip flexion, internal rotation, and adduction compared with the preoperative status [19]. In a comparison study of treatments for symptomatic acetabular retroversion, Zurmühle *et al*. showed that anteverting periacetabular osteotomy was more appropriate than acetabular rim trimming through surgical hip dislocation [20].

It is important to improve hip instability caused by posterior deficiency when accompanied by acetabular retroversion. Capsular repair alone may not provide joint stability, especially in a young patient with a dysplastic acetabulum and a very active lifestyle. Strong posterior and lateral bone support may be necessary to ensure stability of a dysplastic hip with recurrent hip dislocation [6]. This approach yielded excellent results in this study, with the patient actively wakeboarding at 1 year of follow-up.

Therefore, we emphasize that it is important to improve anterior over-coverage and to obtain adequate posterior bone support to prevent recurrent dislocation of the hip. We performed anteverting ERAO for retroversion of the acetabulum related to recurrent posterior dislocation. Both preoperative three-dimensional surgical planning software and an intraoperative navigation system can provide a highly accurate map for this complicated surgery that simultaneously improves the pincer-type FAI and posterior deficiency of the acetabulum. Moreover, this software and navigation system made it possible to improve the postoperative ROM of the hip.

## Conclusions

We performed anteverting ERAO using preoperative three-dimensional surgical planning software and an intraoperative computerized navigation system to treat acetabular retroversion accompanied by recurrent posterior dislocation of the hip. These techniques can provide a highly accurate map for this complicated surgery that simultaneously improves the pincer-type FAI and posterior deficiency of the acetabulum. To the best of our knowledge, this is the first report to describe the treatment of recurrent posterior dislocation of the hip associated with acetabular retroversion using preoperative three-dimensional surgical planning software, an intraoperative computerized navigation system, and postoperative three-dimensional evaluation.

## Abbreviations

AASA: Anterior acetabular sector angle
CT: Computed tomography
DICOM: Digital Imaging and Communications in Medicine
ERAO: Eccentric rotational acetabular osteotomy
FAI: Femoroacetabular impingement
JOA: Japanese Orthopaedic Association
PASA: Posterior acetabular sector angle
ROM: Range of motion
